# Auditory cortical deactivation during speech production and following speech perception: an EEG investigation of the temporal dynamics of the auditory alpha rhythm

**DOI:** 10.3389/fnhum.2015.00534

**Published:** 2015-10-08

**Authors:** David Jenson, Ashley W. Harkrider, David Thornton, Andrew L. Bowers, Tim Saltuklaroglu

**Affiliations:** ^1^Department of Audiology and Speech Pathology, University of Tennessee Health Science CenterKnoxville, TN, USA; ^2^Department of Communication Disorders, University of ArkansasFayetteville, AR, USA

**Keywords:** sensorimotor integration, auditory alpha, EEG, dorsal stream, speech-induced suppression

## Abstract

Sensorimotor integration (SMI) across the dorsal stream enables online monitoring of speech. Jenson et al. (2014) used independent component analysis (ICA) and event related spectral perturbation (ERSP) analysis of electroencephalography (EEG) data to describe anterior sensorimotor (e.g., premotor cortex, PMC) activity during speech perception and production. The purpose of the current study was to identify and temporally map neural activity from posterior (i.e., auditory) regions of the dorsal stream in the same tasks. Perception tasks required “active” discrimination of syllable pairs (/ba/ and /da/) in quiet and noisy conditions. Production conditions required overt production of syllable pairs and nouns. ICA performed on concatenated raw 68 channel EEG data from all tasks identified bilateral “auditory” alpha (α) components in 15 of 29 participants localized to pSTG (left) and pMTG (right). ERSP analyses were performed to reveal fluctuations in the spectral power of the α rhythm clusters across time. Production conditions were characterized by significant α event related synchronization (ERS; *p*FDR < 0.05) concurrent with EMG activity from speech production, consistent with speech-induced auditory inhibition. Discrimination conditions were also characterized by α ERS following stimulus offset. Auditory α ERS in all conditions temporally aligned with PMC activity reported in Jenson et al. (2014). These findings are indicative of speech-induced suppression of auditory regions, possibly via efference copy. The presence of the same pattern following stimulus offset in discrimination conditions suggests that sensorimotor contributions following speech perception reflect covert replay, and that covert replay provides one source of the motor activity previously observed in some speech perception tasks. To our knowledge, this is the first time that inhibition of auditory regions by speech has been observed in real-time with the ICA/ERSP technique.

## Introduction

Human communication relies heavily on the functional integrity of the auditory system. Auditory cortical regions reside bilaterally in the temporal lobes, extending posteriorly along the superior temporal gyri (STG) to include primary and association regions. These regions allow humans to sense sounds and are particularly tuned to speech, providing spectro-temporal analysis of complex acoustic speech signals (Specht, 2014). Auditory association regions, such as the posterior superior temporal gyri (pSTG), comprise the posterior aspects of the dorsal stream, which function in tandem with anterior regions (e.g., the premotor cortex, PMC) of this network to facilitate sensorimotor integration (SMI) for speech (Hickok and Poeppel, 2000, 2004, 2007; Rauschecker, 2012). While there is clear evidence of dorsal stream activity in both speech perception and production (Numminen and Curio, 1999; Callan et al., 2000; Curio et al., 2000; Houde et al., 2002; Herman et al., 2013; Jenson et al., 2014), its temporal dynamics are still not well understood.

According to contemporary models of speech such as the Directions into Velocities of Articulators (DIVA; Tourville and Guenther, 2011; Guenther and Vladusich, 2012) and State Feedback Control (SFC; Hickok et al., 2011; Houde and Nagarajan, 2011), SMI for speech production is dependent upon the integrity of the dorsal stream network. When motor commands for speech are initiated, the PMC produces a corollary discharge (i.e., efference copy) containing an internal model of the expected sensory consequences of the movement (von Holst, 1954; Blakemore et al., 2000; Wolpert and Flanagan, 2001). The efference copy is sent from the PMC to higher order association regions for comparison with available acoustic reafferent feedback, delivered with the execution of the motor commands (Guenther, 2006; Tourville and Guenther, 2011; Guenther and Hickok, 2015). Any mismatch between prediction and reafference (i.e., an error signal) quickly results in corrective feedback sent to motor planning regions (e.g., PMC) for online updating of subsequent commands (Guenther et al., 2006; Houde and Chang, 2015). However, during continuous error-free unperturbed speech production, internally based predictions match the reafferent feedback, minimizing the need for corrective feedback. This accurate matching is thought to have a subtractive (i.e., canceling) effect, producing a net attenuation of activity in auditory regions, which is paramount to distinguishing our own speech from that of others (Blakemore et al., 2000; Wolpert and Flanagan, 2001). This suppression of predicted feedback is thought to enhance sensitivity to deviations from the intended production, facilitating online monitoring of speech (Niziolek et al., 2013; Sitek et al., 2013). This proposal is supported by evidence of lowered auditory thresholds to self-produced vs. externally produced sound (Reznik et al., 2014).

Evidence of speech-induced suppression (SIS; Curio et al., 2000; Sitek et al., 2013) has been demonstrated using various neuroimaging techniques. Positron electron tomography (PET) studies have shown reduced STG activation during speech production compared to listening to playback of one’s own speech (Frith et al., 1991; Hirano et al., 1997; Wise et al., 1999). Similarly, in ERP studies, the amplitude of the N100/M100 response has been found to be reduced in normal overt speech compared to replay tasks (Curio et al., 2000; Houde et al., 2002; Greenlee et al., 2011; Chang et al., 2013), and when compared to speech under altered auditory feedback conditions (Heinks-Maldonado et al., 2005; Behroozmand and Larson, 2011; Kort et al., 2014). Using electrocorticography (ECoG), Chang et al. (2013) found suppression of the pSTG in speaking vs. listening conditions. Taken together, these PET, ERP, and ECoG studies support the deactivation of posterior dorsal stream auditory regions via efference copy during normal speech production in accord with DIVA (Tourville and Guenther, 2011; Guenther and Vladusich, 2012) and SFC (Hickok et al., 2011; Houde and Nagarajan, 2011) models. While the pSTG appears to be the primary site of posterior dorsal stream activity in speech, some studies have reported similar activity in the posterior MTG (Christoffels et al., 2007; Herman et al., 2013; Bowers et al., 2014). Functional magnetic resonance imaging (fMRI) has also produced results that are consistent with these models, identifying suppression in the pSTG during overt speech production (Christoffels et al., 2011). However, some fMRI studies have produced conflicting results. For example, Reznik et al. (2014) reported enhanced responses in the auditory cortex (i.e., pSTG) to self-generated sounds contrasted with externally-generated sounds, interpreting this enhancement as evidence of efference copy improving perceptual sensitivity. Other studies have reported auditory suppression to self-generated stimuli in anterior (i.e., medial) locations of the STG while observing enhancement in posterior regions (Agnew et al., 2013). These mixed findings have been interpreted as representing two functionally distinct and spatially differentiated processes (Agnew et al., 2013; Chang et al., 2013; Houde and Chang, 2015), resolvable with the superior spatial resolution of fMRI.

Though there is ample evidence for SIS around posterior dorsal regions in speech production, a better understanding of its functional role is likely to be achieved by temporally mapping activity within the dorsal stream regions in reference to speech events. Increased temporal precision also may enhance understanding of the functional role of dorsal stream activity observed during speech perception. While dorsal stream activity is not typically observed during “passive” listening tasks (Scott et al., 2009; Szenkovits et al., 2012; Bowers et al., 2013), it has been reported in a variety of more challenging “active” perception tasks, such as discrimination of foreign phonemes (Callan et al., 2006), segmentation (Burton et al., 2000; LoCasto et al., 2004), and discrimination of speech in noise (Bowers et al., 2013, 2014). These mixed findings leave unanswered questions regarding the extent to which auditory-to-motor mapping functionally supports accurate perception vs. being merely a by-product of increased processing. One way to address these questions is by examining the timing of dorsal stream activity relative to stimulus presentation. Early activity may be indicative of predictive coding (Sohoglu et al., 2012), in which early motor representations are used to constrain the analysis of incoming sensory information to aid accurate discrimination; a form of experience-based Constructivist hypothesis testing (Stevens and Halle, 1967; Callan et al., 2010; Skipper, 2014). In contrast, late activity following stimulus offset might reflect covert replay (Burton et al., 2000; Rogalsky et al., 2008; Jenson et al., 2014), in which stimuli are covertly rehearsed in working memory to facilitate discrimination (Baddeley, 2003). Thus, fine-grained temporal data are critical to addressing the functional role of dorsal stream activity in speech perception.

The excellent temporal and spectral detail inherent to neural oscillations makes them a prime candidate for evaluating the dynamics of neural activity in anterior and posterior regions of the dorsal stream (e.g., PMC, pSTG). These oscillations originate from local synchrony between the action potentials of neurons, giving rise to neuronal assemblies with periodic variations in their activity levels (Schnitzler and Gross, 2005; Buszaki, 2006). Fluctuations in spectral power relative to baseline within frequency bands can be measured as relative increases (event related synchronization, ERS) and decreases (event related desynchronization, ERD) in activity, respectively. The “gating by inhibition” hypothesis (Jensen and Mazaheri, 2010) proposes that spectral power within the alpha (α) band (8–13 Hz) can be interpreted as a measure of cortical activation. In support of this hypothesis, α spectral power has been shown to be inversely correlated with the fMRI BOLD signal (Laufs et al., 2003; Brookes et al., 2005; Scheeringa et al., 2009; Mayhew et al., 2013), leading to the interpretation of α ERS and ERD as indices of cortical inhibition and disinhibition, respectively (Klimesch et al., 2007; Weisz et al., 2011).

α oscillations are ubiquitous across the brain, having been implicated in the modulation of cortical activity found in both attention (Muller and Weisz, 2012; Frey et al., 2014) and working memory tasks (Jokisch and Jensen, 2007; van Dijk et al., 2010). A growing body of evidence also points to the existence of an independent auditory α rhythm distinct from other known α generators (Tiihonen et al., 1991; Lehtela et al., 1997; Weisz et al., 2011). Tiihonen et al. (1991) identified a magnetoencephalographic (MEG) α rhythm that demonstrated ERD during auditory stimulation that was not modulated by opening the eyes or clenching the fist, concluding that this was a distinct auditory α rhythm (Tiihonen et al., 1991; Lehtela et al., 1997). Subsequent investigation has implicated this auditory α rhythm in top-down attentional control during dichotic listening (Muller and Weisz, 2012), neural excitability and stimulus detection (Weisz et al., 2014), and auditory hallucinations (Weisz et al., 2011). These studies demonstrate the utility of auditory α oscillations to the investigation of cognitive processes underlying speech.

The temporal precision, economy, and non-invasive nature of electroencephalography (EEG) makes it well suited for capturing oscillatory activity from SMI in speech perception and production (Cuellar et al., 2012; Bowers et al., 2013; Jenson et al., 2014). However, historically EEG analysis has been limited by poor spatial resolution due to volume conduction (the fact that each channel contains information from multiple neural sources) and its susceptibility to contamination by movement artifact. Recently, independent component analysis (ICA) has offered an effective means of overcoming these limitations. ICA is a method of blind source separation that decomposes complex mixtures of non-neural (i.e., artifact) and neural EEG signals into temporally independent and spatially fixed components (Stone, 2004; Onton et al., 2006). In effect, ICA provides a means of both separating muscle movement from neural activity and reliably identifying cortical sources of activity. Independent EEG components can be further decomposed across time and frequency via event related spectral perturbations (ERSP) to reveal patterns of ERS/ERD that characterize regional neural activity in cognitive and motor tasks. Identification of auditory α rhythm components can be followed by ERSP analysis to better understand auditory activity across the time course of speech production and perception tasks.

This ICA/ERSP analysis is well established in perception tasks (Lin et al., 2007, 2011; McGarry et al., 2012) and has more recently been applied to speech perception, examining changes in spectral activity in the sensorimotor μ rhythm components (Bowers et al., 2013, 2014; Jenson et al., 2014). Consistent with Callan et al. (2010) and constructivist interpretations of predictive coding and analysis-by-synthesis (Stevens and Halle, 1967), Bowers et al. (2013) found active syllable discrimination produced more robust mu (μ) ERD (indicating sensorimotor processing) than passive listening to syllables or discriminating between tones. In addition to identifying sensorimotor μ components, Bowers et al. (2014) reported bilateral components from posterior superior temporal lobes (pSTG) with characteristic α spectra, similar to those described by Muller and Weisz (2012). Though ICA clearly has demonstrated the capacity for identifying sources of neural activity in perceptual tasks, its application to motor tasks has been limited due to questions pertaining to its ability to accurately localize and estimate cortical activity within neural sources in the presence of competing myogenic activity (Oken, 1986; Shackman et al., 2009; McMenamin et al., 2010, 2011).

Recently, Jenson et al. (2014) used an ICA/ERSP technique to measure anterior dorsal stream activity in speech perception and production. Specifically, they identified μ components with characteristic α and beta (β; ~20 Hz) peaks and related the changes in spectral power within these peaks to sensory (α) and motor (β) contributions to anterior dorsal stream activity in various tasks. Participants listened to passive noise, discriminated pairs of syllables with and without background noise, and performed overt productions of syllable pairs and tri-syllable nouns. ICA of concatenated raw EEG data from all (perception and production) tasks yielded independent left and right μ components localized to the PMC common to all conditions, supporting the use of ICA in speech production. The ERSP analysis revealed concurrent α and β ERD (reflecting sensory and motor processing) time-locked to muscle movement during overt production. The authors interpreted these findings as evidence of a normal continuous sensorimotor loop for speech production. Interestingly, this same pattern of concurrent α and β μ ERD was observed in the discrimination conditions in the time period following acoustic offset. The authors cautiously interpreted this μ ERD in discrimination conditions as evidence of late covert rehearsal while the stimuli was being held in memory prior to a response (Burton et al., 2000; Baddeley, 2003). This interpretation supports the suggestion that similar anterior dorsal stream sensorimotor processes can be involved in covert and overt speech production (Gehrig et al., 2012; Ylinen et al., 2014), However, to achieve a better understanding of dorsal stream activity in speech perception and production, it is necessary to also examine the temporal dynamics of sensorimotor activity in the posterior aspects of the network.

The purpose of the current study is twofold. The first is to use ICA of raw EEG data to identify temporal lobe auditory components common to speech perception and production tasks. The second is to use ERSP analysis to provide high-resolution temporal and spectral information regarding the dynamics of this auditory α rhythm during speech perception and production. It is hypothesized that ICA will identify bilateral components with α spectra (~10 Hz) localized to auditory association regions, representing activity within posterior regions of the dorsal stream. The second hypothesis is that ERSP analysis of these components will reveal α ERS, representing reduced activity in posterior aspects of the dorsal stream (i.e., pSTG) by an efference copy while speech is being produced. Though the current study employs no connectivity measures, ERSPs from auditory components can be examined alongside those from anterior sensorimotor μ components reported in Jenson et al. (2014) to better understand dorsal stream activity in speech perception and production. Thus, the third hypothesis is that μ ERD and α ERS will be observed simultaneously reflecting synchronous complementary activity across anterior and posterior aspects of the dorsal stream. Observing this pattern of activity following speech perception will support the theory that dorsal stream activity in speech discrimination is characterized at least in part by covert replay.

## Materials and Methods

### Participants

Twenty-nine right-handed native English speakers were recruited from the audiology and speech pathology program at the University of Tennessee Health Science Center. Subjects (24 females, 5 males) had a mean age of 25.16 years (range 21–46) and no history of cognitive, communicative, or attentional disorders. The Edinburgh Handedness Inventory (Oldfield, 1971) was administered to establish handedness dominance for each subject. The Institutional Review Board for the University of Tennessee approved this study, and all subjects provided informed consent prior to participation.

### Stimuli

#### Perception

Syllable stimuli (/ba/ and /da/) for the active perception conditions were generated with AT&T naturally speaking text-to-speech software, which utilizes synthetic analogs of a male speaker. Syllable stimuli were combined to create syllable pairs such that half of the stimuli consisted of identical pairs (e.g., /ba ba/) and half of the stimuli contained different pairs (e.g., /da ba/). Syllable pairs were then low pass filtered at 5 kHz and normalized for root-mean-square (RMS) amplitude. Each syllable was 200 ms in duration and paired syllables were separated by 200 ms, yielding stimuli that were 600 ms from onset of the first syllable to offset of the second syllable.

One of the active perception conditions (discrimination in noise—Ndis) required subjects to discriminate syllable pairs embedded in white noise with a signal-to-noise ratio (SNR) of +4 dB. This condition was included as previous studies have reported that this SNR produces increased dorsal stream activity while allowing participants to accurately discriminate between the syllables (Binder et al., 2004; Osnes et al., 2011; Bowers et al., 2013, 2014). Another discrimination condition (quiet discrimination—Qdis) required participants to discriminate syllable pairs in the absence of background noise. In order to control for a discrimination response bias (Venezia et al., 2012), an equal number of different and identical syllable pairs were used in each discrimination condition. Discrimination responses were made using a button press. The stimulus used for the control (passive listening) condition was continuous white noise.

#### Production

**Figure 1:**
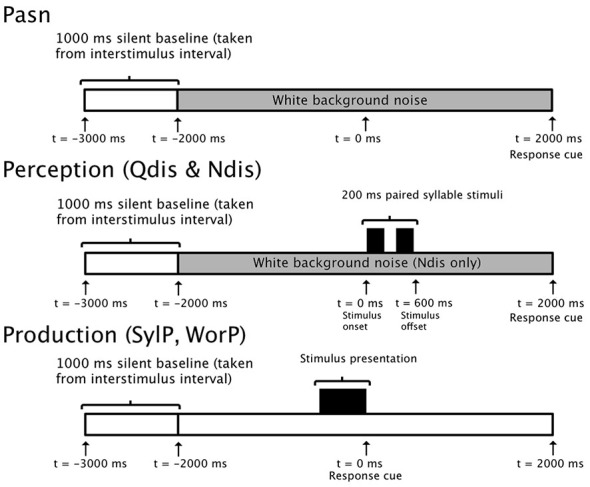
**5000 ms epoch timelines for single trials in control (PasN), discrimination (Qdis and Ndis), and production (SylP and WorP) conditions**.

Targets for speech production consisted of the same syllable pairings used in the discrimination conditions (e.g., /ba da/), as well as tri-syllable nouns initiated with either /b/ or /d/ and followed by a vowel (e.g., buffalo, daffodil). Visual stimuli for production were presented at the center of the visual field on Microsoft PowerPoint slides consisting of white text on a black background (Arial font) subtending a visual angle of 1.14°. The timelines for perception and production tasks are illustrated in Figure 1.

### Design

The experiment consisted of a five condition, within subjects design. The conditions were designed to require gradually increased motoric demands, progressing from the perception of white noise to the production of tri-syllable nouns. The five conditions were:

1. Passive listening to white noise (PasN);
2. Discrimination of syllable pairs in quiet (Qdis);
3. Discrimination of syllable pairs in background noise (Ndis);
4. Overt production of syllable pairs (SylP); and
5. Overt production of tri-syllable nouns (WorP).

Condition 1 was a passive perception task, conditions 2–3 were active perception tasks, and conditions 4–5 were production tasks. The PasN condition required no discrimination, but was used as a control task for the Qdis and Ndis conditions. To control for the neural activity related to the button press response required in the Qdis and Ndis conditions, a button press response was used in the PasN condition. Conditions 2–4 used paired /ba/ and /da/ syllables. Qdis and Ndis required active discrimination of syllable pairs, while SylP required overt productions of /ba/ /da/ syllable pairs, respectively. The WorP condition required overt production of tri-syllable nouns initiated by a /b/ or /d/ and followed by a vowel. Stimuli for the WorP condition were selected from Blockcolsky et al. (2008).

### Procedure

The experiment was conducted in an electrically and magnetically shielded, double-walled, soundproof booth. Participants were seated in a comfortable chair with their head and neck supported. Stimuli were presented and button press responses were recorded by a PC computer running Compumedics NeuroScan Stim 2, version 4.3.3. A button press response was embedded in the PasN condition for two reasons: (1) to control for anticipatory β suppression which has been previously reported in tasks requiring a button press response (Makeig et al., 2004; Graimann and Pfurtscheller, 2006; Hari, 2006) and (2) requiring a button press response in a condition with no active discrimination ensured that the subjects were attending to and engaged in the task. The response cue for all perception conditions was a 100 ms, 1000 Hz tone presented at the end of the trial epoch (2000 ms post stimulus). In the PasN condition, subjects were instructed to sit quietly, listen to the stimulus (i.e., white noise), and press the button when they heard the response cue. In the Qdis and Ndis conditions, subjects were instructed to press one of two buttons after hearing the response cue depending on whether the syllables were judged to be the same or different. Handedness of button press response was counterbalanced across all subjects and conditions. Discrimination accuracy was determined as percentage of trials correctly discriminated, and subjects who did not discriminate at a level significantly above chance were excluded from the analysis.

In the production conditions, visual stimuli were presented on a monitor (69.5 × 39 cm) placed 132 cm in front of the participant’s chair. Visual stimuli (syllable pairs and words) remained on the screen for 1 s, and participants were instructed to begin their production when the visual stimuli disappeared. Thus, stimulus offset was the response cue in the production conditions. In the SylP and WorP conditions, subjects were instructed to produce the syllable pairs in their normal speaking voice. All productions were complete within the 2000 ms window between the response cue and the end of the trial epoch. Each of the five conditions was comprised of 2 blocks of 40 trials each, yielding a total of 10 blocks (5 conditions × 2 blocks). Order of block presentation was randomized for each subject.

#### EEG Acquisition

Whole head EEG data were acquired from 68 channels. These channels included two electromyography (EMG) and two electrocardiogram (EKG) electrodes. Data were recorded with an unlinked, sintered NeuroScan Quik Cap, based on the extended international standard 10–20 system (Jasper, 1958; Towle et al., 1993). All recording channels were referenced to the linked mastoid channels (M1, M2). The electro-oculogram was recorded by means of two electrode pairs placed above and below the orbit of the left eye (VEOL, VEOU) and on the medial and lateral canthi of the left eye (HEOL, HEOR) to monitor vertical and horizontal eye movement. The two EMG electrodes were placed above and below the lips to capture labial lip movement related to overt speech.

EEG data were recorded using Compumedics NeuroScan Scan 4.3.3 software in tandem with the Synamps 2 system. EEG data were band pass filtered (0.15–100 Hz) and digitized with a 24-bit analog to digital converter with a sampling rate of 500 Hz. Data collection was time locked to stimulus onset in the perception conditions, and to the response cue in the production conditions. The visual stimuli to be produced were displayed on the screen for 1 s prior to disappearing, which served as the response cue for production. Thus, time zero was defined as stimulus onset for the perception conditions, and stimulus offset served as time zero for the production conditions.

#### EEG Data Processing

Data processing and analysis were performed with EEGLAB 12 (Brunner et al., 2013), an open source MATLAB toolbox. Data were processed at the individual level and analyzed at both the individual and group level. The following steps were performed at each stage:

1. Individual processing/analysis:
  (a) Preprocessing of 10 raw EEG files for each participant (5 conditions × 2 blocks);
  (b) ICA of preprocessed files across conditions for each participant; and
  (c) Localization of all neural and non-neural dipoles for each independent component.

2. Group analysis:
  (a) Two separate analyses using the STUDY module of EEGLAB 12; one study targeting neural components only (“in-head”) and the other targeting neural and myogenic components (“all”);
  (b) Components common across participants clustered by means of Principal Component Analysis (PCA);
  (c) Left and right STG clusters identified from the “in-head” STUDY analysis, while peri-labial EMG cluster was identified from the “all” STUDY;
  (d) Left and right STG clusters localized by equivalent current dipole (ECD) and current source density (CSD) methods; and
  (e) ERSP performed to yield time-frequency analysis of activity in STG and EMG clusters.

### Analysis for Hypothesis 1

#### Data Preprocessing

Raw EEG data files from both blocks of each condition were appended to create one dataset per condition per participant, and then downsampled to 256 Hz to reduce the computational requirements of further processing steps. Trial epochs of 5000 ms (ranging from −3000 to +2000 ms around time zero) were extracted from the continuous EEG data. The data were then filtered from 3–34 Hz, which allowed for clear visualization of α and β bands, while filtering muscle artifact from surrounding frequency bands. All EEG channels were referenced to the mastoids (M1, M2) to remove common mode noise. Trials were visually inspected, and all epochs containing gross artifact (in excess of 200 μV) were removed. Additionally, trials were rejected if the participant performed the discrimination incorrectly, or if the response latency exceeded 2000 ms. A minimum of 40 useable trials per subject per participant was required in order to ensure a successful ICA decomposition.

#### ICA

Following data preprocessing and prior to ICA analysis, data files for each participant were concatenated to yield a single set of ICA weights common to all conditions. This allowed for comparison of activity across conditions within spatially fixed components. The data matrix was decorrelated through the use of an extended Infomax algorithm (Lee et al., 1999). Subsequent ICA training was accomplished with the “extended runica” algorithm in EEGLAB 12 with an initial learning rate of 0.001 and the stopping weight set to 10–7. ICA decomposition yielded 66 ICs for each participant, corresponding to the number of recording electrodes (68 data channels–2 reference channels; M1, M2). Scalp maps for each component were generated by projecting the inverse weight matrix (W-1) back onto the original spatial channel configuration.

After ICA decomposition, equivalent current dipole models (ECD) were generated for each component by using the boundary element model (BEM) in the DIPFIT toolbox, an open source MATLAB plugin available at sccn.ucsd.edu/eeglab/dipfit.html (Oostenveld and Oostendorp, 2002). Electrode coordinates conforming to the standard 10–20 configuration were warped to the head model. Automated coarse-fitting to the BEM yielded a single dipole model for each of the 1914 ICs (29 participants × 66 ICs). Dipole localization entails a back projection of the signal to a potential source that could have generated the signal, followed by computing the best forward model from that hypothesized source that accounts for the highest proportion of the scalp recorded signal (Delorme et al., 2012). The residual variance (RV) is the mismatch between the original scalp recorded signal and this forward projection of the ECD model. The RV can be interpreted as a goodness of fit measure for the ECD model.

#### STUDY (Group Level Analyses)

Group level analyses were performed in the EEGLAB STUDY module. The STUDY module allows for the comparison of ICA data across participants and conditions. The STUDY module also allows for the inclusion or exclusion of ICs based on RV and location (in head vs. outside head). Two different STUDY analyses were performed on participants’ ICA files containing dipole information. The “in head” analysis (neural) was limited to dipoles originating within the head, and the RV threshold was set to <20%.

In order to capture peri-labial EMG activity, a second STUDY (“all”) was performed, which included dipoles originating both within the head and outside the head. Additionally, the RV threshold was lifted to <50% to account for the fact that EMG activity inherently contains higher levels of RV. Peri-labial EMG activity was extracted from the “all” STUDY, while all neural data were analyzed within the “in head” study only.

#### PCA Clustering

In both of the STUDY analyses (“in head” and “all”), IC pre-clustering was performed based on commonalities of spectra, dipoles, and scalp maps. The K-means statistical toolbox was used to group similar components across participants based on the specified criteria via PCA. ICs from the “in head” analysis were assigned to 25 neural clusters, from which left and right auditory clusters were identified. ICs from the “all” analysis were assigned to 66 possible clusters (both neural and non-neural), one of which contained peri-labial EMG activity.

Designation to auditory (STG) clusters for the “in head” STUDY was based primarily on the initial results of PCA, followed by inspection of all ICs in the auditory cluster and surrounding clusters based on spectra, dipoles, and scalp maps. Inclusion criteria for the auditory clusters were based on previously observed posterior dorsal stream activity in speech and, therefore, included components that were localized to the pSTG or pMTG regions, showed a characteristic α spectrum, and could be localized with RV <20%.

The majority of the 66 clusters generated in the “all” STUDY contained non-neural (myogenic) activity. The cluster containing peri-labial EMG activity was identified based on dipole location and verified by ERSP analysis, demonstrating activity during the overt speech conditions only.

#### Source Localization

Source localization for ECD clusters identified in the STUDY module is the mean of the Talairach coordinates (*x, y, z*) for each of the contributing dipole models (identified by the DIPFIT module). A further method of source localization is standardized low-resolution brain electromagnetic tomography (sLORETA), which addresses the inverse problem by using CSD from scalp recorded electrical signals to estimate source location (Pascual-Marqui, 2002). Solutions are based on the Talairach cortical probability brain atlas, digitized at the Montreal Neurological Institute (MNI). Electrode locations are co-registered between both spherical models (BESA) and realistic head geometry (Towle et al., 1993). The 3-D brain space was divided into 6,239 voxels, yielding a spatial resolution of 5 mm. The inverse weight projections from the original EEG channels for each component contributing to the temporal α clusters were exported to sLORETA. Cross-spectra were computed and mapped to the Talairach atlas and cross-registered with MNI coordinates, resulting in CSD estimates for each contributing component. The analysis of statistical significance of CSD estimates across participants was performed in the sLORETA software package. The analysis was non-parametric, based on the estimation (via randomization) of the probability distribution of the t-statistic expected under the null hypothesis (Pascual-Marqui, 2002). This method corrects for multiple comparisons across all voxels and frequencies (3–34 Hz). Voxels that were significant at *p* < 0.001 were considered to be active across participants. Group level source localizations are based on the CSD source estimates computed via sLORETA, though the ECD localizations are also reported as they serve to demonstrate the inter-subject variability present in the data.

### Analysis for Hypotheses 2 and 3

#### ERSP

ERSP analyses were used to measure fluctuations in spectral power (in normalized decibel units) across time in the frequency bands of interest (3–34 Hz). Time-frequency transformations were computed using a Morlet wavelet rising linearly from three cycles at 3 Hz to 34 cycles at 34 Hz. Trials were referenced to a 1000 ms pre-stimulus baseline selected from the inter-trial interval. A surrogate distribution was generated from 200 randomly sampled latency windows from this silent baseline (Makeig et al., 2004). Individual ERSP changes across time were calculated with a bootstrap resampling method (*p* < 0.05 uncorrected). Single trial data for all experimental conditions for frequencies between 4 and 30 Hz and ranging from −500 to 1500 ms were entered into the time-frequency analysis.

In the “in head” study, permutation statistics (2000 permutations) were used to assess inter-condition differences. The significance threshold was set at *p* < 0.05, and Type 1 error was controlled by false discovery rate (FDR) correction (Benjamini and Hochberg, 2000). Statistical analyses used a 1 × 5 repeated measures ANOVA design (PasN, Qdis, Ndis, SylP, WorP). Further *post hoc* analyses of differences in perception and production conditions used 1 × 3 and 1 × 2 repeated measures ANOVA designs, respectively.

## Results

### Discrimination Accuracy

All subjects that contributed to the temporal α clusters performed the discrimination tasks with a high degree of accuracy. As it has been shown that activity in sensorimotor regions is susceptible to the effects of response bias (Venezia et al., 2012), d’ values also are reported to tease out the differential effects of sensitivity and response bias on perceptual accuracy. The average number of useable trials (out of 80) for each condition was: PasN = 74.4 (SD 6.9), Qdis = 74 (SD 4.77), Ndis = 69.1 (SD 12.52), SylP = 73.73 (SD 5.19), and WorP = 73.14 (SD 6.39). Subjects performed the discrimination with similar high accuracy in both the Qdis [96.5%, SD 2.55; d’ 3.38, SE 0.09] and Ndis [94.5, SD 8.69; d’ 3.63, SE 0.19] conditions. The greater variability in the Ndis condition was due primarily to one participant, who performed the task with 65% accuracy. A paired *t*-test on d’ values for each condition indicated that subject accuracy for these two conditions was not significantly different (*p* > 0.05). The mean reaction time for discrimination conditions was 506.3 ms in the Qdis conditions (SD 133.3) and 568.1 ms in the Ndis condition (SD 298.3). A paired *t*-test indicated that the mean response latency between conditions was not significantly different (*p* > 0.05). Taken together, these findings indicate that subjects performed both discrimination tasks with similar levels of accuracy and efficiency. A response contingent analysis was performed in which incorrectly discriminated trials were excluded from subsequent analysis, and thus the analysis of neural data was restricted only to those associated with correctly discriminated trials.

### Results Pertaining to Hypothesis 1

#### Temporal α Cluster Characteristics

**Figure 2:**
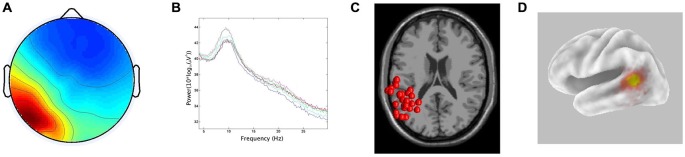
**Results for left temporal cluster. (A)** Scalp distribution, scaled in root-mean-square (RMS) microvolts, **(B)** Mean spectra of cluster components, **(C)** equivalent current dipole (ECD) localization for cluster components demonstrating inter-subject variability, **(D)** current source density (CSD) cluster localization projected onto a van Essen cortical model. Active voxels are significant at *p* < 0.001 (corrected for multiple comparisons).

**Figure 3:**
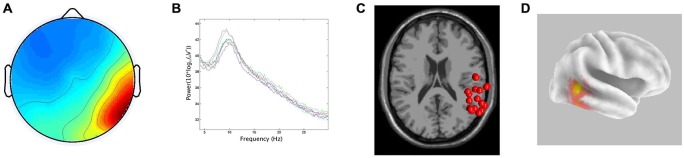
**Results for right temporal cluster. (A)** Scalp distribution, scaled in RMS microvolts, **(B)** Mean spectra of cluster components, **(C)** ECD localization for cluster components demonstrating inter-subject variability, **(D)** CSD cluster localization projected onto a van Essen cortical model. Active voxels are significant at *p* < 0.001 (corrected for multiple comparisons).

In line with the hypothesis that ICA would identify bilateral α components localized to pSTG, 15/29 participants generated components with less than 20% RV contributing to both the left and right temporal α clusters. The clusters had peaks at 10 Hz on both the left and right. For the ECD dipole models, the average dipole localization was at Talairach [−48, −45, 15] for the left temporal cluster and Talairach [57, −42, 10] on the right. The percentage of unexplained variance for these two clusters was 11.7% and 11.9%, respectively. The CSD model computed with sLORETA showed active voxels (*p* < 0.001) localized to the pSTG on the left and the pMTG on the right. In both hemispheres, activation spread across the pSTG and pMTG. CSD source maxima were localized to MNI [−50, −55, 10] on the left and MNI [55, −45, 0] on the right. The Euclidean distance between ECD and CSD sources were 11.4 mm on the left and 10.6 mm on the right. The peri-labial EMG cluster, identified on the basis of dipole location and the time course of activity, consisted of non-neural components with an average of 20.07% RV. Figures 2, 3 demonstrate: (A) the average scalp map; (B) the spectra; (C) the distribution of ECD dipoles; and (D) CSD source localization for the left and right temporal clusters, respectively. As the component activations were generated from data concatenated across conditions, the source localizations reported pertained to temporal lobe clusters from all experimental conditions.

### Results Pertaining to Hypothesis 2

#### ERSP Analysis in Production (SylP, WorP)

**Figure 4:**
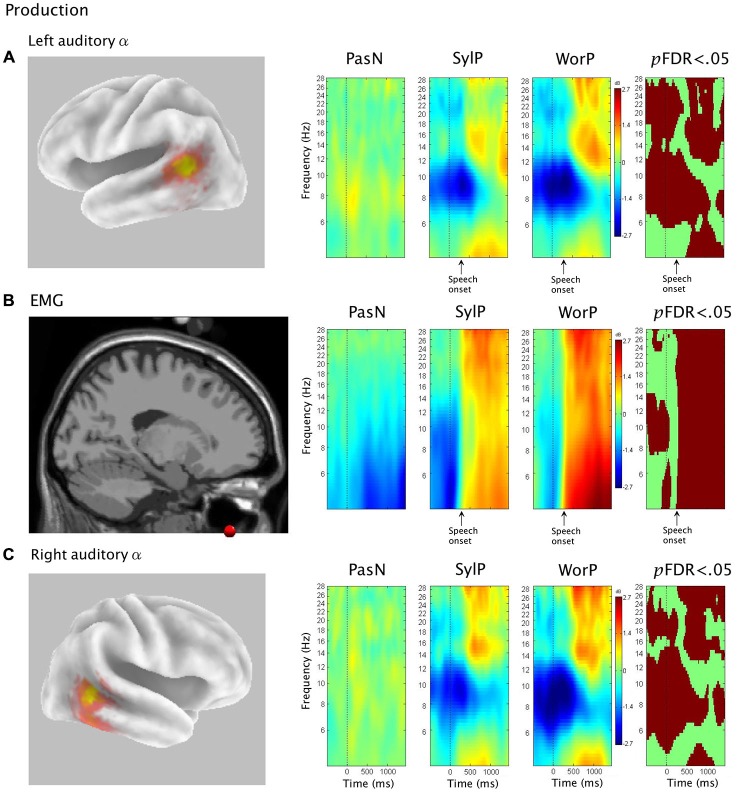
**The first column in rows (A) and (C) show sLORETA source solutions for left and right temporal α clusters, respectively, while the first column in row (B) shows the mean ECD localization of the peri-labial EMG cluster**. The middle columns show event related spectral perturbation (ERSP) analyses, mapping ERS and ERD in the control condition (PasN) and production conditions (SylP and WorP). The far right column shows significant time-frequency differences in spectral power across conditions (*p* < 0.05, corrected for multiple comparisons). Time courses across ERSP analyses were identical, though rows **(A,B)** reference speech onset while row **(C)** references the timeline from Figure 1.

The second hypothesis was that ERSP analysis of auditory α clusters would reveal α ERS in time periods coinciding with overt production. Figure 4 shows van Essen maps (computed with sLORETA) demonstrating activated voxels at (*p* < 0.001) in the (A) left and (C) right hemisphere temporal clusters. ERSP analyses show differential patterns of ERS/ERD measured against baseline across the two production conditions (SylP, WorP), within the 4–30 Hz bandwidth. The final column shows significant differences (*p*FDR < 0.05) compared to PasN. Figure 4B shows the average ECD localization for the EMG cluster corresponding to peri-labial muscle activity, as well as the ERSP analysis of that component cluster.

In the left temporal cluster, α ERD in production conditions (SylP, WorP) began prior to stimulus onset, peaking after the cue to produce speech. Approximately 500 ms after the production cue, α ERD began to decrease accompanied by the emergence of α ERS, which extended into low β frequencies. As in perception conditions, the right temporal cluster showed identical patterns of activation, though with weaker spectral power.

#### Temporal Alignment Between Temporal α, Sensorimotor μ, and Peri-labial EMG Activity

EMG activity was found in the SylP and WorP conditions only. EMG ERS (corresponding to speech production) began at approximately 300 ms and peaked at about 500 ms post response cue. These response latencies are within the expected range for speech production tasks (Heinks-Maldonado et al., 2005). α ERS in left and right temporal clusters was aligned temporally with EMG ERS in the SylP and WorP conditions.

**Figure 5:**
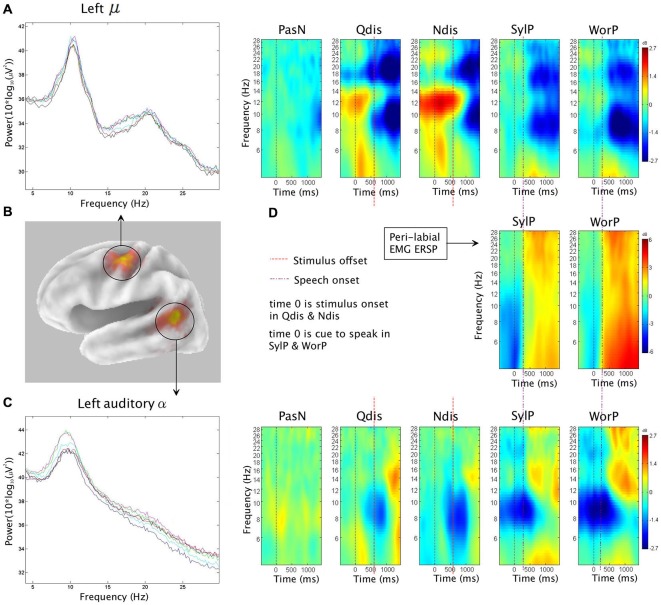
**The first column shows (B) sLORETA source solutions for the sensorimotor μ (reproduced from Jenson et al. (2014) with permission) and temporal α clusters, as well as mean spectra for (A) the sensorimotor μ cluster and (C) the temporal α cluster**. Subsequent columns show ERSP analyses depicting patterns of corresponding ERS and ERD in the neural and **(D)** peri-labial myogenic clusters. In production conditions (SylP and WorP), the onset of peak peri-labial EMG activity **(D)** temporally aligns with the emergence of sensorimotor μ ERD **(A)** and temporal α ERS **(C)**. In perception conditions (Qdis and Ndis), the emergence of sensorimotor μ ERD **(A)** temporally aligns with the emergence of temporal α ERS **(C)**.

Jenson et al. (2014) analyzed data from the same subject pool in identical conditions and interpreted concurrent α and β ERD over the PMC as evidence of covert replay during perception and overt production of speech. In the current study, the emergence of α ERS in the temporal cluster also was aligned temporally with the peak α and β ERD in the sensorimotor μ rhythm reported by Jenson et al. (2014) in both perception and production. Figure 5 demonstrates the temporal synchrony between α ERS in the temporal lobe, sensorimotor α/β ERD (representing SMI during production), and EMG ERS, as well as the alignment of temporal α ERS and sensorimotor α/β ERD (consistent with covert rehearsal) during discrimination tasks.

### Results Pertaining to Hypothesis 3

#### ERSP Analysis in Perception (PasN, Qdis, Ndis)

**Figure 6:**
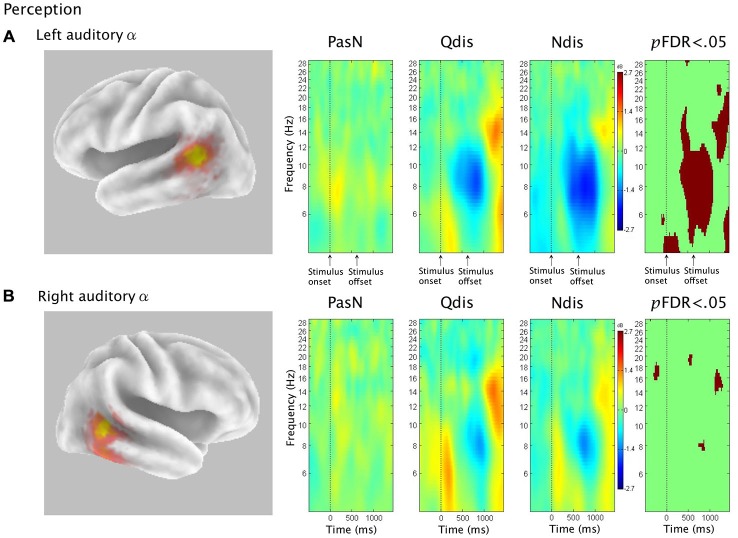
**Rows (A,B) show sLORETA source solutions for left and right temporal α clusters, respectively, followed by ERSP analyses from passive listening (PasN) and active discrimination conditions (Qdis, Ndis) displaying patterns of ERS and ERD relative to baseline in the middle columns**. The far right column shows significant time-frequency differences in spectral power across conditions (*p* < 0.05, corrected for multiple comparisons). Time courses across ERSP analyses were identical, though row **(A)** references stimulus onset and offset while row **(B)** references the timeline from Figure 1.

The third hypothesis was that ERSP analysis of auditory α clusters in discrimination conditions would demonstrate α ERS during time periods of μ ERD, consistent with the interpretation of PMC activity during speech discrimination as evidence of covert replay. Figure 6 shows van Essen maps (computed with sLORETA) showing activated voxels (*p* < 0.001) in the left (A) and right (B) hemisphere temporal clusters. ERSP analyses show differential patterns of ERS/ERD measured against baseline across the three perception conditions (PasN, Qdis, Ndis), within the 4–30 Hz bandwidth. The final column shows significant differences (*p*FDR < 0.05) among the three conditions.

For the left temporal cluster, α ERD began subsequent to acoustic stimulation and persisted until approximately 500 ms post stimulus offset. At approximately 500 ms post stimulus offset, α ERD began to decrease, giving way to α ERS in both discrimination conditions (Qdis and Ndis) that extended into low β frequencies. A *post hoc* comparison of the two discrimination conditions (Qdis and Ndis) revealed no significant differences between conditions. The right hemisphere temporal cluster showed the same patterns of α ERD fading to ERS as the left temporal cluster, though with weaker spectral power. *Post hoc* comparisons of Qdis and Ndis to the PasN condition produced identical results to those found in the left temporal cluster.

## Discussion

The first hypothesis (that ICA would identify bilateral α clusters localized to auditory association regions) was well supported by the data obtained from the left hemisphere. This finding is consistent with the MEG/EEG findings of Weisz et al. (2011) who also found evidence of an independent auditory α rhythm. Clusters of neural activity emanating from these regions with <20% unexplained RV were localized to the pSTG via both ECD and CSD localization methods (although their exact source averages varied by ~1 cm). This localization is in agreement with previous findings of auditory α oscillatory activity identified via EEG/ICA (Bowers et al., 2014) and MEG (Muller and Weisz, 2012) and is consistent with a left-hemisphere dominance for dorsal stream activity in speech-based tasks (Hickok and Poeppel, 2004). In the right hemisphere, the two localization techniques also produced source averages that were separated by ~1 cm. However, the average ECD source was in the pSTG, while the average CSD source was located slightly inferiorly in the pMTG, possibly highlighting the uncertainty of EEG source localization and a reduced role of the right hemisphere in speech processing.

The finding that only 15/29 participants contributed to the clusters requires examination. Reasons for this include (1) the application of a standard head model reducing localization accuracy; (2) the location of auditory regions along the Sylvian fissure. As anatomic variability increases at greater distances from midline, the potential impact of a standard head model may have been maximal along the lateral and dorsal surfaces of the STG; (3) EEG’s superior sensitivity to signals arising from cortical gyri rather than sulci; and (4) the fact that only components from pSTG and pMTG regions were included though all participants produced temporal lobe components. It should be noted that α activity has been observed across more anterior portions of the STG in addition to the pSTG (Weisz et al., 2011). However, due to the possibility that anterior and posterior regions of the STG perform functionally distinct tasks (Agnew et al., 2013; Chang et al., 2013; Houde and Chang, 2015) and that the current goal was to examine dorsal stream activity, only components localized to posterior regions were included in this study. Therefore, it is likely that these inclusion criteria limited the number of contributors to the clusters. Even with the inherent limitations in source localization, the ERSP analyses produced significant changes in α spectral power across time in both speech perception and production conditions, providing evidence of auditory cortical deactivation that can be interpreted in light of current models.

### Auditory α ERS/ERD in Speech Production

Auditory α activity in both production conditions (SylP, WorP) was characterized by ERD prior to production with no significant differences in activity between the two conditions. Before initiating speech, participants read the target and prepared to speak while attending to the visual cue to do so. Early activation of the auditory cortex (following stimulus presentation and prior to production) has previously been demonstrated during silent and overt reading (Kell et al., 2011). Reduced levels of α activity in sensory regions prior to stimulus presentation have been shown to facilitate detection of near-threshold stimuli in the somatosensory domain (Weisz et al., 2014), and a similar mechanism in the auditory domain may facilitate monitoring of speech by an SMI loop. Additionally, α ERD also is known to result from simple increases in attention (Jensen and Mazaheri, 2010). Therefore, considering that the current speech production tasks required the coordination of multiple cognitive processes prior to speaking, all of which rely on attention to some extent, it is not possible to parse out the individual contributions of all cognitive processes to ERD prior to speech. Rather, it is likely that pre-speech α ERD resulted from contributions of attention, reading, and integration of auditory regions into an error-free SMI loop for speech.

The hypothesis that speech production would be characterized by increases in oscillatory α power was supported. A positive shift in auditory α power emerged concurrently with robust peri-labial EMG activity (i.e., muscle movement) that marked the initiation of speech. These patterns of neural and muscular activity were observed after ~300 ms (i.e., reaction time) from the cue to speak (time 0, Figure 4). It should be noted that these ERS/ERD changes were measured in reference to a “silent baseline” prior to each trial and that they were statistically significant when compared to a control passive listening condition in which little pSTG α activity was observed. As α ERS is associated with reduced activity (Laufs et al., 2003; Brookes et al., 2005; Scheeringa et al., 2009; Mayhew et al., 2013), the current findings are consistent with speech-induced suppression (i.e., modulation of auditory cortical activity during speech production; Frith et al., 1991; Hirano et al., 1997; Curio et al., 2000; Houde et al., 2002; Heinks-Maldonado et al., 2005; Christoffels et al., 2007, 2011). However, it is also interesting to note that though auditory oscillatory activity was characterized by α spectra, the observed ERS spread into higher frequencies and may be somewhat consistent with ECoG and fMRI studies that have implicated modulations in auditory gamma frequencies during speech production (Greenlee et al., 2011; Agnew et al., 2013; Reznik et al., 2014).

It is also important to note that both myogenic and auditory activity during speech production is characterized by ERS, which may raise questions pertaining to the possibility of muscle activity contaminating neural activity. There are multiple reasons to refute this notion. First, if ICA was not able to adequately separate neural signals from myogenic artifact, muscle activity would have overwhelmed the α and β ERD recorded in the sensorimotor μ cluster (Jenson et al., 2014). Second, α ERS was noted in the perception conditions (coinciding with periods of covert rehearsal—see below), during which no overt response was required. Together, this evidence suggests that α ERS resulted from neural activity as opposed to myogenic artifact.

The larger picture of dorsal stream activity in speech production becomes apparent when the current data are viewed alongside those of Jenson et al. (2014). Data from the same participants in the same conditions showed sensorimotor μ ERD (i.e., disinhibition) beginning with muscle movement in speech production. Thus, when viewed together, sensorimotor disinhibition and auditory inhibition coincided with speech production (as indicated by EMG activity; see Figure 5). Current models of SMI for speech indicate that the sensorimotor loop is initiated by the generation of a motor plan in PMC (Tian and Poeppel, 2010; Houde and Nagarajan, 2011; Jenson et al., 2014). Concurrent with the delivery of this motor plan to primary motor cortex (M1), an efference copy of the expected sensory consequences is sent to auditory regions for comparison with the goals and outcomes of the movements. Any deviations from expectations are detected and corrective feedback is sent to the PMC. As true auditory and somatosensory reafferent feedback is received, this information is also integrated into feedback to the PMC (Tian and Poeppel, 2010). During normal error-free production, articulatory predictions are matched to the available sensory (i.e., auditory) information, resulting in the observed net deactivation in auditory association regions. Based on this model, it was not surprising to see near perfect temporal concordance between α/β μ ERD, auditory α ERS, and peri-labial EMG activity in normal, unperturbed speech production. Thus, the results of the current study demonstrate inhibition of auditory regions during overt speech, consistent with the suppression that would be expected based on the delivery of an efference copy from the PMC.

### Auditory α ERS/ERD in Accurate Speech Discrimination

In both the quiet (Qdis) and noisy (Ndis) discrimination conditions, participants listened to pairs of syllables and then waited ~1400 ms to make an active same/different discrimination response. Considering that only correct responses were analyzed, the following interpretations of the oscillatory data are made in reference to accurate speech discrimination. Auditory activity prior to and during stimulus onset was characterized by α ERD. During this same time period, β ERD was observed within the μ components localized to anterior regions of the dorsal stream (e.g., PMC; Jenson et al., 2014). This pattern of PMC β ERD activity has previously been explained as early predictive coding (i.e., hypothesis generation via internal modeling) followed by hypothesis testing via auditory to motor integration (Alho et al., 2014), according to analysis by synthesis theories (Stevens and Halle, 1967). The current data from auditory regions which indicate increased auditory activity prior to and during stimulus presentation continue to support this interpretation, though it is necessary to consider how a predictive coding explanation might be favored over one of simple attention, which has also been known to modulate β activity in cognitive tasks (van Ede et al., 2014). Participants were briefed on the task prior to each discrimination condition and therefore knew what to expect. In addition, only four syllable pairs were possible. Therefore, across 80 trials per condition, it is likely that participants were able to formulate general internal models of the expected stimuli (i.e., syllables) to help constrain the upcoming sensory analysis. Further support for speech-related predictive coding comes from Bowers et al. (2013), who reported early μ β ERD in similar syllable but not tone discrimination tasks.

The time period following stimulus offset and prior to the response was characterized by temporal α ERS, similar to that observed in the production conditions. Jenson et al. (2014) found sensorimotor μ ERD in the same time period. They interpreted this as evidence of covert rehearsal, during which the syllables were held in working memory to facilitate accurate discrimination and response. The current findings support this explanation. Clearly, it can be seen in Figure 5 that in both Qdis and Ndis conditions, sensorimotor μ ERD is aligned temporally to auditory α ERS; a pattern similar to that observed in the production conditions. It should be noted that covert rehearsal has been used to explain motor activity sometimes observed in speech perception tasks (Burton et al., 2000; Baddeley, 2003; Jenson et al., 2014; Roa Romero et al., 2015). However, this assertion lacks support without temporal data showing when activity occurred relative to stimulus onset and offset. By demonstrating anterior sensorimotor disinhibition aligned with auditory inhibition following stimulus offset in the absence of peri-labial EMG activity, these data support the theory that covert rehearsal can account for some of the motor activity observed during accurate speech discrimination tasks such as these (Burton et al., 2000; Wilson et al., 2004; Callan et al., 2006, 2010; Bowers et al., 2013; Jenson et al., 2014). However, it should be noted, as indicated above, that covert rehearsal may not be the only explanation for this activity. Prior to and during syllable discrimination there is evidence of anterior sensorimotor activity characterizing internal modeling (Bowers et al., 2013, 2014; Jenson et al., 2014).

While the presence of anterior dorsal stream activity during covert production is relatively well established (Neuper et al., 2006; Gehrig et al., 2012; Jenson et al., 2014), it remains unclear how a subtractive mechanism in posterior dorsal stream regions could function in the absence of re-afferent feedback. It has been proposed that auditory inhibition is linked to the delivery of an efference copy. As no overt production took place during the discrimination conditions, it may seem surprising that the auditory cluster should demonstrate α ERS during covert rehearsal as the observed auditory suppression is thought to be contingent on efference copy delivery linked to motor plan execution. These findings are not without precedent, however, as lip-reading (Kauramäki et al., 2010; Balk et al., 2013) and covert speech (Tian and Poeppel, 2015) have been shown to reduce auditory cortical responses. There is also evidence that failure of this sensory suppression in covert productions may be associated with some of the positive symptoms (i.e., auditory hallucinations) of schizophrenia (Ford et al., 2001; Ford and Mathalon, 2004, 2005), indicating that auditory suppression during covert production is critical to normal function. One possible explanation for auditory suppression when efference copy delivery is questionable is that in the absence of an efference copy, auditory suppression may be based on higher order processes (Crapse and Sommer, 2008). In line with this explanation, sensory inhibition has recently been linked to motor intention prior to overt activity (Stenner et al., 2014). Additionally, it is possible that during covert production, auditory suppression may be based on the comparison of sensory predictions to a higher-level sensory goal. This is consistent with a recent proposal by Skipper (2014), who suggested that the auditory hypotheses being evaluated are internal in nature based on context and prior experience rather than being dependent on available acoustic information. However, further investigation is warranted to better explain how auditory inhibition can occur during covert speech processing.

### Summary

These results illustrate how fluctuations in oscillatory power in time characterize posterior dorsal stream activity across speech perception and production. Viewing these data alongside activity from anterior dorsal regions (Jenson et al., 2014; Figure 5) provides a window for understanding the temporal dynamics of dorsal stream activity in speech discrimination and production events. Prior to speech production, the pSTG is active (as evidenced by α ERD) and primed to receive speech. In this experiment, α ERD occurred as participants read the stimuli to be produced. As speech was initiated, anterior dorsal regions (e.g., PMC, μ ERD) became active as activity in the pSTG was attenuated (α ERS). The patterns of oscillatory activity across these cortical regions aligned temporally with muscle activity and are suggestive of auditory suppression arising from an efference copy driven sensorimotor loop that enables online monitoring during normal speech production (Guenther et al., 2006; Tian and Poeppel, 2010, 2015; Hickok et al., 2011; Houde and Nagarajan, 2011; Arnal and Giraud, 2012; Hickok, 2012). However, this interpretation is based solely on descriptions of the strength and timing of activity across regions and should be made with caution. Connectivity measures across these regions are necessary to provide more direct evidence of the efference copy mechanism.

Perhaps not surprisingly, dorsal stream activity in accurate speech discrimination is more complex. Prior to stimulus onset, anterior dorsal regions (e.g., PMC) are active, most likely reflecting the recruitment of motor/attentional mechanisms for internal modeling that help constrain the ensuing auditory analysis. Both anterior and posterior regions of the dorsal stream become active while stimuli are presented (evident by μ ERD), most likely indicative of hypothesis testing (analysis by synthesis). Finally, following stimulus offset and in the absence of reafferent feedback from overt production, activity in anterior dorsal regions is further enhanced (μ ERD), while activity in posterior regions (temporal α ERS) is suppressed. This pattern of late dorsal stream activity is similar to that observed during speech production and indicative of covert rehearsal following stimulus offset, potentially driven by efference copy. Based on these findings, it is feasible that the dorsal stream plays a variety of roles across the time course of stimuli expectancy, presentation, and rehearsal to facilitate accurate perception. However, because there were insufficient data from inaccurate discrimination trials for comparison, it is not currently possible to determine the extent to which each of these processes individually contributes to perceptual acuity. Oscillatory fluctuations reflecting activation changes across the time course of speech discrimination suggests a dynamic rather than static role for the dorsal stream. Activity before, during, and after stimulus presentation may be explained as internal modeling, analysis by synthesis (or perhaps direct realism), and covert production, respectively. Taken together, the results converge on a dynamic constructivist perspective espousing the notion that speech discrimination is facilitated by embodied articulatory representations, attention, experience, and short-term memory (Callan et al., 2006, 2010, 2014; Galantucci et al., 2006; Bowers et al., 2013, 2014; Jenson et al., 2014).

### Limitations and Future Directions

While the results of this study provide compelling evidence that the neural dynamics of the temporal α oscillator and the sensorimotor μ rhythm work in synchrony to accomplish online monitoring of speech in production and hypothesis testing in perception, certain limitations should be addressed. The source localization of auditory α clusters should be interpreted with caution based on the inherent uncertainty of source localization when performing EEG with 68 electrodes. In this study, the uncertainty was illustrated by the difference between ECD and CSD source localizations. The hypothesized communication between these two clusters of independent components in this study is based purely on temporally aligned concordant patterns of ERS/ERD. While it is clear that these regions are co-active in time periods that could support a sensorimotor feedback loop, direct transfer of information cannot be inferred solely on the basis of the data presented. Direct evidence for cortico-cortical communication between these two regions during speech perception and production requires further analysis with a measure that is able to assess coherence between cortical regions (e.g., any frequency-sensitive variant of Granger causality). However, such connectivity analyses are beyond the scope of this paper. In addition, analyses in the current study were restricted (according to our hypotheses) to activity in the α band, which characterized the spectrum of the components in the region of interest. However, there was also evidence of differential activity in other frequencies (e.g., theta-gamma nesting; Giraud and Poeppel, 2012), though their analysis was beyond the scope of the current study.

It should also be noted that the tasks used in this study (discrimination and production of syllable pairs in isolation) may not be representative of normal human communication (Hickok and Poeppel, 2000; Skipper, 2014). They lack normal audiovisual and semantic contextual cues, potentially requiring greater levels of processing than would be required in normal communicative situations. Despite these potential shortcomings, the temporal alignment of sensorimotor μ ERD, peri-labial EMG activity, and temporal α ERS strongly suggests the presence of a sensorimotor feedback loop for online monitoring and hypothesis testing, and warrants further investigation with methods able to establish cortico-cortical communication. Finally, deeper understanding of typical sensorimotor activity for speech enables the analysis and description of neural activity in clinical populations such as individuals who stutter, in whom auditory regions are found to show even greater deactivation than normal, possibly due to compromised SMI during speech production (Max et al., 2003; Brown et al., 2005; Watkins et al., 2008).

## Conclusion

ICA identified components over auditory association cortices with expected characteristic α spectra. ERSP analysis of temporal α components demonstrated reduced activity concurrent with periods of overt and covert production. These findings demonstrate the utility of the ICA/ERSP analysis for localizing and temporally delineating neural activity in speech events. The temporal alignment of auditory α ERS, sensorimotor μ ERD, and peri-labial EMG activity in production tasks supports previous interpretations of temporal α ERS indexing a relative deactivation of auditory regions which may possibly be attributed to an efference copy mechanism involved in the online monitoring of ongoing speech. In perception conditions, the synchrony of temporal α ERS and sensorimotor μ ERD likely represent a similar mechanism as subjects engaged in covert rehearsal of syllable pairs held in working memory. These observed phenomena reflecting the interactions of multiple dorsal stream regions may provide a framework for describing normal speech-related sensorimotor activity. The non-invasive and cost-effective nature of the technique supports its continued application to investigating neural network dynamics in normal and clinical populations of all ages.

## Conflict of Interest Statement

The authors declare that the research was conducted in the absence of any commercial or financial relationships that could be construed as a potential conflict of interest.
